# Case report: Novel SIN3A loss-of-function variant as causative for hypogonadotropic hypogonadism in Witteveen–Kolk syndrome

**DOI:** 10.3389/fgene.2024.1354715

**Published:** 2024-03-11

**Authors:** Lourdes Correa Brito, Ana Keselman, Florencia Villegas, Paula Scaglia, María Esnaola Azcoiti, Sebastián Castro, Nora Sanguineti, Agustín Izquierdo, Marianela Maier, Ignacio Bergadá, Claudia Arberas, Rodolfo A. Rey, María Gabriela Ropelato

**Affiliations:** ^1^ Centro de Investigaciones Endocrinológicas “Dr. César Bergadá” (CEDIE), CONICET—FEI—División de Endocrinología, Hospital de Niños Ricardo Gutiérrez, Buenos Aires, Argentina; ^2^ Sección Genética Médica, Hospital de Niños Ricardo Gutiérrez, Buenos Aires, Argentina; ^3^ Unidad de Medicina Traslacional, Hospital de Niños Ricardo Gutiérrez, Buenos Aires, Argentina; ^4^ Departamento de Biología Celular, Histología, Embriología y Genética, Facultad de Medicina, Universidad de Buenos Aires, Buenos Aires, Argentina

**Keywords:** hypogonadotropic hypogonadism, Kallmann syndrome, *SIN3A* gene, Witteveen–Kolk syndrome, whole-exome sequencing, case report

## Abstract

Pubertal delay can be due to hypogonadotropic hypogonadism (HH), which may occur in association with anosmia or hyposmia and is known as Kallmann syndrome (OMIM #308700). Recently, hypogonadotropic hypogonadism has been suggested to overlap with Witteveen–Kolk syndrome (WITKOS, OMIM #613406) associated with 15q24 microdeletions encompassing *SIN3A*. Whether hypogonadotropic hypogonadism is due to haploinsufficiency of *SIN3A* or any of the other eight genes present in 15q24 is not known. We report the case of a female patient with delayed puberty associated with intellectual disability, behavior problems, dysmorphic facial features, and short stature, at the age of 14 years. Clinical, laboratory, and imaging assessments confirmed the diagnosis of Kallmann syndrome. Whole-exome sequencing identified a novel heterozygous frameshift variant, NM_001145358.2:c.3045_3046dup, NP_001138830.1:p.(Ile1016Argfs*6) in *SIN3A*, classified as pathogenic according to the American College of Medical Genetics and Genomics (ACMG/AMP) criteria. Reverse phenotyping led to the clinical diagnosis of WITKOS. No other variant was found in the 96 genes potentially related to hypogonadotropic hypogonadism. The analysis of the other contiguous seven genes to *SIN3A* in 15q24 did not reveal any clinically relevant variant. In conclusion, these findings point to *SIN3A* as the gene in 15q24 related to the reproductive phenotype in patients with overlapping WITKOS and Kallmann syndrome.

## 1 Introduction

Pubertal delay is defined as the lack of occurrence of secondary sexual characteristics at an age that is at least two standard deviations later than the population mean, as defined by Marshall and Tanner (1969, 1970). This means the absence of thelarche, that is, the appearance of the breast bud, at the age of 13 years in girls or of testicular volume increase, that is, >3 mL, at the age of 14 years in boys (Palmert and Dunkel, 2012). Amongst the genetic disorders underlying pubertal delay, hypogonadotropic (or central) hypogonadism (HH) explains 3.2% of the cases in girls and 13.3% in boys (Jonsdottir-Lewis et al., 2021). Hypogonadotropic hypogonadism may occur isolated or in association with anosmia or hyposmia, known as Kallmann syndrome (OMIM #308700) (Dwyer et al., 2023). The advent of high-throughput sequencing techniques has led to the understanding of the genetic basis of hypogonadotropic hypogonadism in patients with complex syndromes, such as CHARGE syndrome (OMIM #214800) due to *CHD7* variants, Waardenburg syndrome (OMIM #613266) associated with *SOX10* mutations, TUBB3 syndrome (OMIM #600638) due to E410K mutation in *TUBB3* (Dentici et al., 2020), and Pallister–Hall syndrome (OMIM #146510) caused by mutations in *GLI3* (Grinspon, 2022). In many of these syndromes, the reproductive phenotype remains underdiagnosed, with the clinical focus driven to the complex symptomatology. Recently, it has been suggested that hypogonadotropic hypogonadism may overlap with Witteveen–Kolk syndrome (WITKOS, OMIM #613406) (Liu and Mapow, 2020; Schnoll et al., 2023).

WITKOS is associated with heterozygous loss-of-function (LOF) variants in switch-independent 3 transcription regulatory family member A (*SIN3A*) (OMIM *607776) or 15q24 microdeletions encompassing *SIN3A*. This autosomal dominant disorder is characterized by a broad spectrum of neurological and physical phenotypes. Various degrees of intellectual disability, neurodevelopmental disorders, behavioral problems, and neurological symptoms have been well-established in the early descriptions of the syndrome (Witteveen et al., 2016). Typical facial dysmorphisms have been consistently reported. Additionally, abnormalities in growth, skeletal structure, hearing, visual disorders, gastrointestinal function, and ectodermal features have also been documented in patients with WITKOS (Balasubramanian et al., 2021). Early or late signs of congenital hypogonadotropic hypogonadism, such as micropenis, cryptorchidism, or pubertal delay, have only been reported in patients with 15q24 microdeletions (Liu and Mapow, 2020). However, whether hypogonadotropic hypogonadism is due to haploinsufficiency of *SIN3A* or any of the other eight genes located in the shortest region of overlap (∼260 kb) of 15q24 could not be ascertained (Magoulas and El-Hattab, 2012; Liu and Mapow, 2020). Our aim was to report the case of a girl with WITKOS and congenital hypogonadotropic hypogonadism carrying a pathogenic heterozygous frameshift variant in *SIN3A* and, furthermore, to conduct a literature review to find evidence supporting the hypothesis that hypogonadotropic hypogonadism could be an additional feature within the spectrum of WITKOS.

## 2 Case presentation

The propositus was a 6-year-old girl referred to us for short stature. She was born at 34 weeks by cesarean section due to oligohydramnios and intrauterine growth retardation (IUGR) and small for gestational age [1,510 g, −1.76 standard deviation score (SDS) for Argentine population (Lejarraga et al., 2009)]. She required mechanical ventilation due to respiratory distress syndrome and nutritional recovery after an episode of hypoglycemia in the neonatal period. During follow-up, she required attention for anemia and thrombocytopenia, recurrent bronchiolitis complicated with pneumonia, and febrile seizures. She was the second daughter of healthy non-consanguineous parents, with two siblings and four healthy paternal siblings. There was no family history of developmental delay. Her developmental milestones were delayed with multiple dyslalias and deficit in motor coordination. Based on neurological examinations, she was diagnosed with intellectual disability and began a speech therapy and school integration program. She also showed a behavioral disorder that was characterized as an attention-deficit hyperactivity disorder (ADHD).

At first visit, she presented with microcephaly (47.5 cm, −3.00 SDS), long face with a high anterior hairline, broad forehead, epicanthal folds, strabismus, wide medial eyebrows, wide nasal base with prominent nasal bridge, retrognathia, short and smooth philtrum, ears with overfolded helix and large lobes, hands with thin digits and bilateral radial deviation of the second digits, clinodactyly of the fifth finger, and joint laxity. Further assessment revealed sensorineural hearing impairment and a bicuspid aortic valve.

**FIGURE 1:**
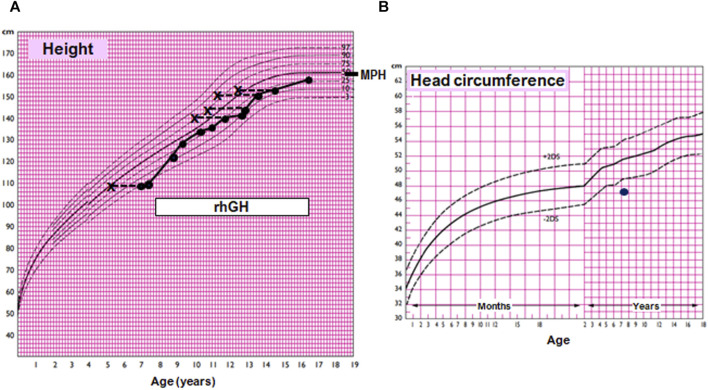
Growth and head circumference charts for the female population according to Argentine standards. **(A)** Growth chart of the index case. MPH, mid-parental adjusted height. The box indicates treatment with recombinant human growth hormone (rhGH). **(B)** Head circumference chart of the index case, showing microcephaly.

**FIGURE 2:**
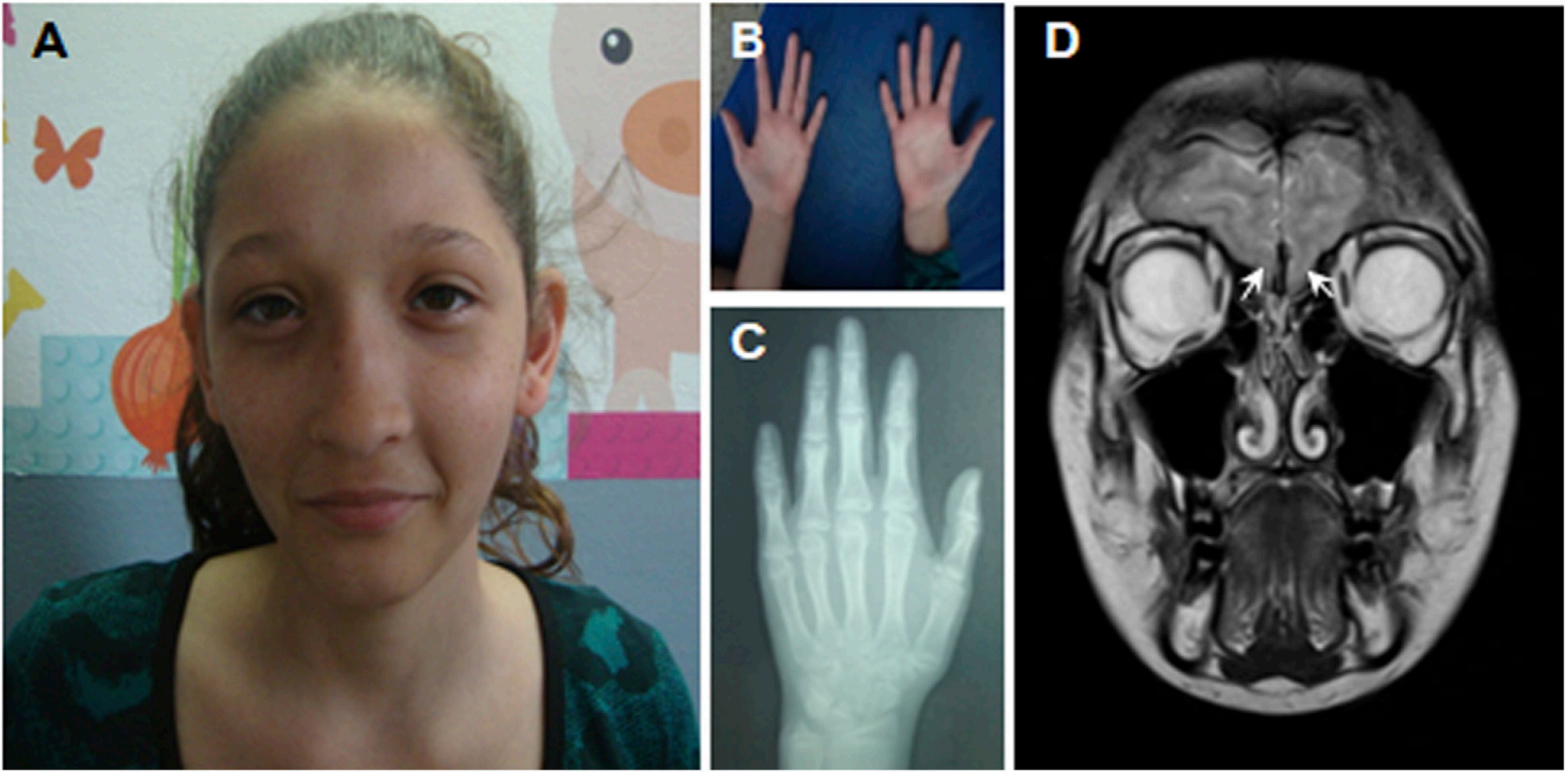
Anatomic features and bone age of the patient at the age of 14 years. **(A)** Microcephaly, long face with a high anterior hairline, broad forehead, epicanthal folds, strabismus, wide medial eyebrows, wide nasal base, prominent nasal bridge, short and smooth philtrum, small mouth, ears with overfolded helix, and large lobes. **(B)** Hands with thin digits and radial deviation of the second digits bilaterally, clinodactyly of the fifth finger. **(C)** Bone age was estimated between 12 and 13 years, according to Greulich and Pyle (1959). **(D)** MRI of the brain showing the absence of the olfactory bulbs (white arrows).

At the age of 6 years 10 months, her height was 109.2 cm (−1.62 SDS), below mid-parental height (164.6 cm, 0.60 SDS), and her weight was 15.4 kg (−2.54 SDS). At 7 years 8 months, her growth velocity was 4.2 cm/year (third centile), and her height SDS was impaired (−2.10 SDS; 110.6 cm). Routine blood tests (RBC, WBC, glucose, BUN, creatinine, SGOT, SGPT, cholesterol, triglycerides, and celiac antibodies) showed normal results. TSH, fT4, IGF1, IGFBP3, cortisol, prolactin, and insulin were within normal ranges. With a diagnosis of small for gestational age without catch-up growth, she was treated with recombinant growth hormone at 0.33 mg/kg/week with a good response: between 8 years 7 months and 11 years 8 months, the height velocity ranged between 5.4 and 7.4 cm/year (Figure 1). At the age of 14 years (Figure 2), she showed the absence of thelarche (Tanner stage M1) and pubarche (PH1); her height was 154 cm (−0.66 SDS); her growth velocity was prepubertal at 4.6 cm/year; and her bone age was delayed (12 years 6 months). She was therefore evaluated for delayed puberty.

## 3 Diagnostic assessment

To evaluate gonadotropin function, a GnRH IV infusion test was performed as previously published (Grinspon et al., 2010). In brief, the patient was subjected to a GnRH IV infusion test (100 μg GnRH, Luteoliberina; Elea SACIFyA, Buenos Aires, Argentina; 0.83 μg/min for 120 min) using the AVI 270 infusion pump (AVI Inc., 3M Healthcare, St. Paul, MN). Serum follicle-stimulating hormone (FSH) and luteinizing hormone (LH) were determined in the serum at 0–120 min and estradiol at baseline in the serum sample. All hormones were measured using validated assays, as previously published (Bergadá et al., 2006). Serum estradiol was undetectable, and gonadotropins were low after the GnRH infusion test (basal LH 0.2 IU/L, peak 0.7 IU/L; basal FSH 0.1 IU/L, peak 0.6 IU/L), leading to the diagnosis of hypogonadotropic hypogonadism. Pelvic ultrasound showed a prepubertal normal uterus, and the ovaries were 1.0 and 0.9 cm^3^. Magnetic resonance imaging (MRI) showed bilateral agenesis of the olfactory bulbs (Figure 2), and anosmia was detected by olfactometry. Altogether, a clinical diagnosis of Kallmann syndrome was reached. Hormone replacement treatment was started gradually with normal evolution. During follow-up, severe scoliosis, aggressive behavior, and ulcerative colitis were found. The final height was 158 cm (−0.4 SDS).

## 4 Timeline of events

**FIGURE 3:**
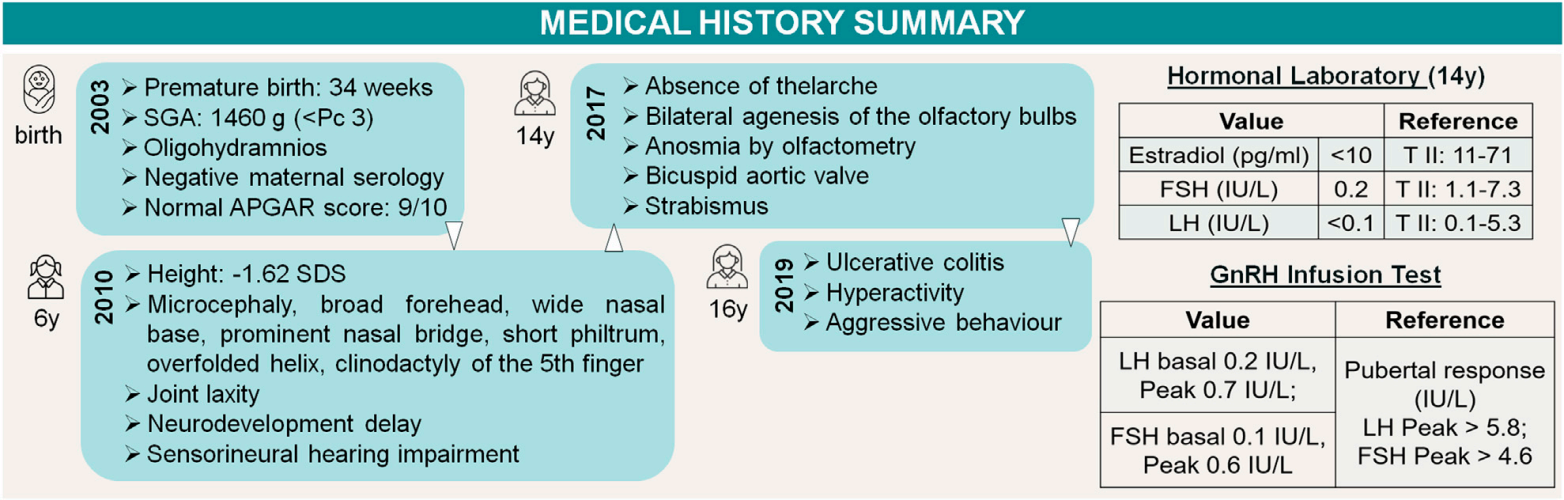
Timeline of events. Patient medical history summary and gonadotropin axis assessment. References available in “3 Diagnostic assessment”.

The patient's medical history summary is shown in Figure 3.

## 5 Genetic diagnosis

The karyotype was 46,XX. To investigate a potential genetic etiology for syndromic hypogonadotropic hypogonadism, a next-generation sequencing (NGS) approach was followed.

### 5.1 NGS and variant filtering and prioritization

The genomic DNA was extracted from the peripheral venous blood cells using the Gentra Puregene Blood Kit (QIAGEN). The DNA was quantified using a high-performance microvolume spectrophotometer NanoPhotometer^®^ NP60 (Implen Inc.), and the DNA concentration was normalized to 10 ng/μL using the Qubit^®^ 3.0 fluorometer (Invitrogen). DNA purity was assessed by measuring the absorbance ratio 260/280 nm; further DNA sample processing was performed only if the ratio was between 1.8 and 2.1. Two different methods for DNA library preparation were performed. At first instance, we used the TruSight One^®^ (TSO) sequencing panel (Illumina), which provides coverage of 4,813 genes associated with known Mendelian genetic disorders (∼12 Mb genomic content, https://www.illumina.com/products/by-type/clinical-research-products/trusight-one.html). The quality of genomic DNA fragmentation was controlled using a capillary system Fragment Analyzer™ (Agilent). Next-generation sequencing by synthesis with fluorescent reversible terminator deoxyribonucleotides was performed using a NextSeq 500^®^ system (Illumina) at the Translational Medicine Unit of the Buenos Aires Children’s Hospital (Unidad de Medicina Traslacional, Hospital de Niños Ricardo Gutiérrez, Buenos Aires). As no variant could be prioritized in the TSO study, subsequently, whole-exome sequencing (WES) was performed by 3billion, Inc. (Seoul, Republic of Korea). All exon regions of all human genes (∼22,000) were captured using xGen Exome Research Panel v2 (Integrated DNA Technologies, Coralville, IA, United States). The captured regions of the genome were sequenced with NovaSeq 6000 (Illumina, San Diego, CA, United States). For the processing of sequencing data from both the TruSight One panel and WES, we followed the best practice recommendations from Broad Institute using the Genome Analysis Toolkit (GATK). The raw sequence data were mapped to the 1000 Genomes phase II reference genome (GRCh37 version hs37d5) using the BWA-MEM algorithm of Burrows–Wheeler Aligner software. Duplicates were removed using Picard (Broad Institute). The variant call format (VCF) file was annotated using ANNOVAR (Wang et al., 2010), and including the ClinVar (https://www.ncbi.nlm.nih.gov/clinvar/), gnomAD (https://gnomad.broadinstitute.org/), and dbSNP databases (https://www.ncbi.nlm.nih.gov/snp/). Variant filtering and prioritization were performed using B_platform (https://www.bitgenia.com/b-platform/). Candidate variants, found in both TSO sequencing panel and WES, were selected when the minor allele frequency (MAF) was <3% in gnomAD exomes and genomes, in 1000 Genomes, and in the Bitgenia database of over 100 Argentine control individuals (http://apps.bitgenia.com/100exomas). Single-nucleotide variants (SNVs) and indels with a read depth ≥10×, genotype quality (GQ) score ≥45, and high/moderate impact on protein were scored based on their association with the patient’s clinical phenotype using the VarElect application (https://varelect.genecards.org/). The following HPO terms were used as input: small for gestational age (HP:0001518), neurodevelopmental delay (HP:0012758), febrile seizures (HP:0002373), inappropriate behavior (HP:0000719), joint hyperflexibility (HP:0005692), short stature (HP:0004322), microcephaly (HP:0000252), broad forehead (HP:0000337), wide nasal base (HP:0012810), curved middle phalanx 5th finger (HP:0009173), hypogonadotropic hypogonadism (HP:0000044), anosmia (HP:0000458), hypoplasia of the olfactory bulb (HP:0040326), sensorineural hearing impairment (HP:0000407), and bicuspid aortic valve (HP:0001647). For further analysis, variants with high and moderate impact on protein among the 96 candidate genes for hypogonadotropic hypogonadism were considered. The list included genes with strong (*ANOS1*, *CHD7*, *FEZF1*, *FGF8*, *FGFR1*, *FSHB*, *GLI2*, *GNRH1*, *GNRHR*, *IL17RD*, *KISS1*, *KISS1R*, *KLB*, *LHB*, *LHX4*, *NR0B1*, *NSMF*, *PROK2*, *PROKR2*, *PROP1*, *SOX10*, *SOX2*, *TAC3*, *TACR3*, and *WDR11*), moderate (*AMH*, *AMHR2*, *AXL*, *CCDC141*, *CUL4B*, *DCAF17*, *DCC*, *DUSP6*, *FGF17*, *FLRT3*, *GLI3*, *HESX1*, *HS6ST1*, *IGSF10*, *LEP*, *LEPR*, *LHX3*, *RNF216*, *SEMA3A*, *SEMA3C*, *SEMA3E*, *SEMA7A*, *SOX3*, *SPRY4*, and *TUBB3*), and limited (*BMP4*, *CPE*, *EBF2*, *GHSR*, *GLCE*, *HAMP*, *HDAC8*, *HFE*, *IGFALS*, *IGSF1*, *MC3R*, *MKRN3*, *MSX1*, *NDN*, *NDNF*, *NELFCD*, *NEMF*, *NR5A1*, *OTX2*, *PAX6*, *PCSK1*, *PHIP*, *PLXNA1*, *RAB18*, *RBM28*, *SLC29A3*, *SLC40A1*, *SMCHD1*, *TCF12*, and *TFR2*) evidence of association with HH, as well as genes recently linked to hypogonadotropic hypogonadism (*CHL1*, *JAG1*, *NHLH2*, *NOTCH1*, *NOTCH2*, *NTN1*, *OTUD4*, *PNPLA6*, *POLR3A*, *POLR3B*, *PTCH1*, *SEMA3F*, *SEMA3G*, *SLIT2*, *SOX11*, and *TBX33*). Integrative Genomics Viewer (IGV v.1.4.2) (Robinson et al., 2011) was used to visually inspect the variants. The Human Genome Variation Society (HGVS) nomenclature was checked using Mutalyzer 3 (Lefter et al., 2021). Pathogenicity predictors, such as CADD (https://cadd.gs.washington.edu/) and REVEL (https://sites.google.com/site/revelgenomics/about), were used to predict variant implications on protein function. The likelihood of nonsense-mediated mRNA decay (NMD) was predicted using the NMDEscPredictor (https://nmdprediction.shinyapps.io/nmdescpredictor/). We classified the variants according to their potential pathogenicity using the ACMG/AMP guidelines for variant interpretation (Richards et al., 2015) and following the ClinGen Sequence Variant Interpretation Working Group (SVI WG) recommendations (https://www.clinicalgenome.org/working-groups/sequence-variant-interpretation). Additionally, CNVs were screened using the coverage-based DECoN (Detection of Exon Copy Number variants) algorithm (Fowler et al., 2016) and analyzed according to ACMG and ClinGen recommendations (Riggs et al., 2020). The details of the filtering and prioritization strategy described above and horizontal coverage data of the hypogonadotropic hypogonadism panel are available in Supplementary Appendices S1, S2.

A total of 69 genes associated with hypogonadotropic hypogonadism were covered to various degrees (Supplementary Appendix S1). We found a variant in *NOTCH1*, which was considered due to its role in the development of kisspeptin neurons (Biehl and Raetzman, 2015). The variant, NM_017617.5:c.3829G>A NP_060087.3:p.(Asp1277Asn) was classified as VUS (score: 0 points = 2P-2B; criteria: PM2_Supp, PP2_Supp, BP4_Mod). In the literature, only one patient with normosmic hypogonadotropic hypogonadism carrying a variant in *NOTCH1* has been reported (PMID: 33208564; https://olida.ibsquare.be/detail/Combination/OLI843/), but with oligogenic inheritance, that is, an additional variant in another gene was required for pathogenicity. Therefore, we considered that the *NOTCH1* variant found in the present case did not have sufficient evidence to be considered as causative of hypogonadotropic hypogonadism. No other relevant variant was found using our filtering protocol (Supplementary Table S1).

**FIGURE 4:**
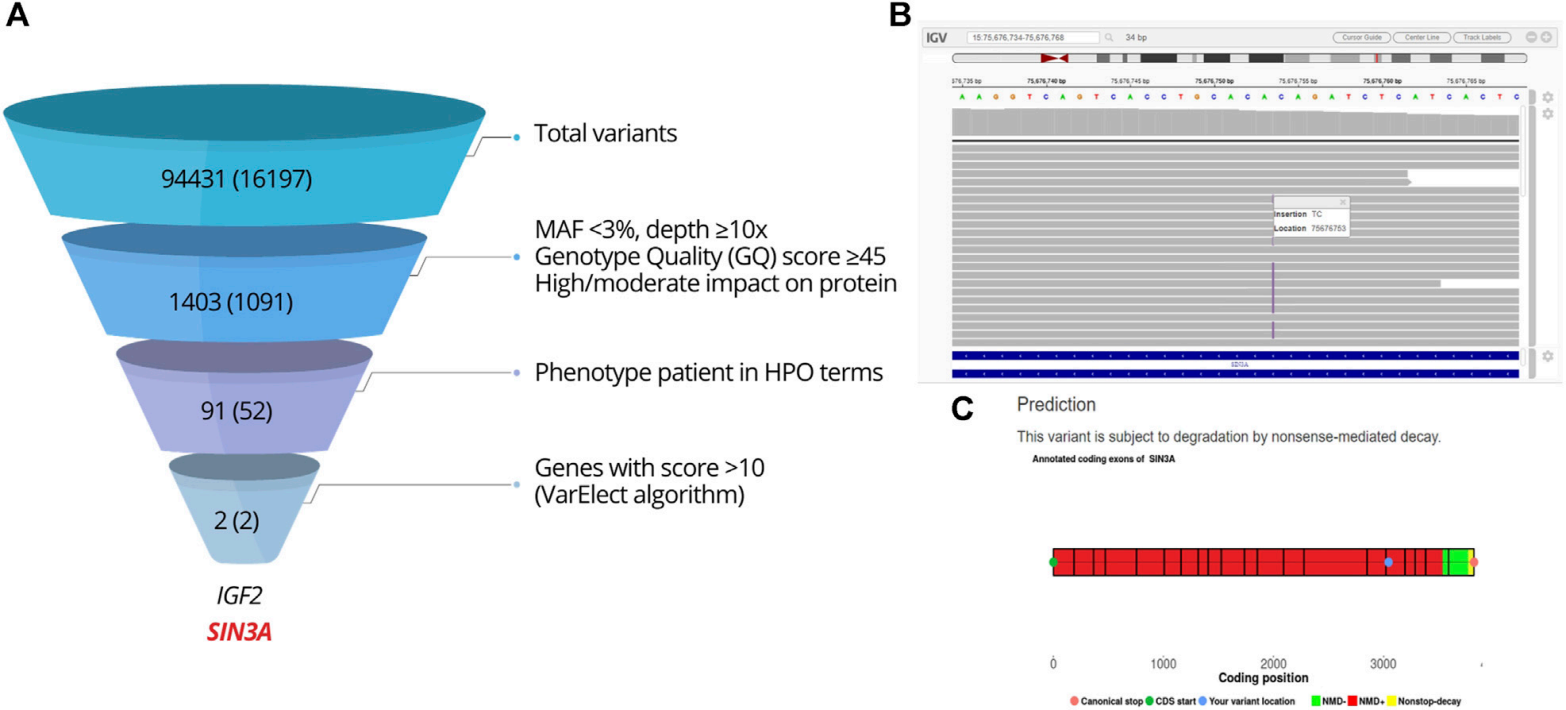
Analysis of the WES result. **(A)** WES filtering and prioritization chart. Steps of the variant filtering protocol in the index case leading to the prioritization of *SIN3A*. The figures indicate the number of variants found, with the number of genes between parentheses. **(B)** Integrative Genomics Viewer (IGV) visualization of variant *SIN3A* NM_001145358.2:c.3045_3046dup, NP_001138830.1:p.(I1016Rfs*6). **(C)** Prediction of NMD using NMDEscPredictor.

Considering the syndromic phenotype associated with hypogonadotropic hypogonadism, the case was submitted to WES, performed by 3billion, Inc. (Seoul, Republic of Korea). The sequencing data are accessible in NCBI's Sequence Read Archive (SRA): https://www.ncbi.nlm.nih.gov/sra/PRJNA1055058. A total of 94,431 variants were detected. Two genes showed a score >10 on VarElect algorithm and were further analyzed (Figure 4). The NM_001127598.3:c.-3-1G>A variant in *IGF2* was classified as likely benign (score: −4 points = 0P-4B; criterion: BS2). A heterozygous frameshift variant in *SIN3A*, at chromosome 15 position 75676753, was prioritized. It was a duplication of one guanine and one adenine in exon 17: NM_001145358.2:c.3045_3046dup. The insertion of the GA dinucleotide predicted a frameshift change [NP_001138830.1:p.(Ile1016Argfs*6)], expected to result in the NMD mechanism or protein truncation (Figure 4).

### 5.2 Sanger sequencing

Sanger sequencing confirmed the presence of the variant in the proband, and its absence in her mother; unfortunately, her father’s DNA was not available. *SIN3A* exon 17 was amplified by polymerase chain reaction (PCR) with the specific primers (forward 5′-AAA​ATG​ATA​CAG​TGG​TTT​GTG​TGA-3′; reverse 5′-TCA​CAG​GTC​AAA​GTA​CCC​CG-3′) and GoTaq^®^ DNA Polymerase (Promega). The products were sequenced using an ABI 3500 Genetic Analyzer (Applied Biosystems) at the Translational Medicine Unit of the Buenos Aires Children’s Hospital (Unidad de Medicina Traslacional, Hospital de Niños Ricardo Gutiérrez, Buenos Aires). The sequences were compared to the reference sequence and analyzed using BioEdit (BioEdit sequence alignment editor) and Chromas (Technelysium Pty Ltd.) tools. The following reference sequences were used: GRCh37 (human genome), *SIN3A*: NG_052855.1 (gene), NM_001145358.2 (mRNA), and NP_001138830.1 (protein).

The variant was not reported in gnomAD exomes and genomes, 1000 Genomes, or in the literature. We reported this variant to ClinVar (VCV001333264.1). The frameshift variant was classified as pathogenic according to the ACMG/AMP and ClinGen SVI WG recommendations (score: 10 points = 10P-0B; criteria: PVS1, PM2_Supp, PP4; Supplementary Table S2). These findings, together with the reassessment of the patient’s phenotype, led to the diagnosis of WITKOS, which explained the short stature in the context of multiple dysmorphic features and neurodevelopmental delay. We asked whether the variant could also explain the Kallmann syndrome (hypogonadotropic hypogonadism) diagnosed in our patient. We first ruled out the existence of clinically significant variants in the 96 genes associated with hypogonadotropic hypogonadism in the WES study (Supplementary Appendix S2). Furthermore, DECoN-CNV prediction algorithm did not detect deletions or duplications in the abovementioned genes. Finally, we scrutinized for pathogenic and probably pathogenic variants in the complete WES data, using InterVar and ClinVar databases. Three rare heterozygous variants were identified in *BCH3*, *TBP*, and *TRDN* genes. The phenotypes associated with these genes were not compatible with those observed in the patient and were discarded as relevant to explain Kallmann syndrome. Finally, we wished to rule out the existence of pathogenic variants in the other seven disease-causing genes present in the shortest region of overlap of 15q24 characteristic of WITKOS. No pathogenic or likely pathogenic variant was found in *COX5A*, *CYP11A1*, *EDC3*, *MAN2C1*, *MPI*, *SEMA7A*, or *STRA6* (Supplementary Table S2).

## 6 Discussion

In this work, we report the genetic diagnosis of a novel heterozygous frameshift pathogenic variant in *SIN3A* in a girl with clinical features of both WITKOS and Kallmann syndrome (hypogonadotropic hypogonadism with anosmia/hyposmia). No other potential candidate variants in the genes associated with Kallmann syndrome were found, and no clinically relevant CNVs were predicted from the WES data.

SIN3A is part of a core histone deacetylase complex, involved in transcriptional silencing mediated through interactions with repressors and corepressors. SIN3–HDAC–MECP2 corepressor complexes play an important role during various phases of embryonic development, such as cell cycle events and cell proliferation (Grzenda et al., 2009). Interestingly, MECP2 has been suggested to participate in the epigenetic control of human pubertal timing (Canton et al., 2023). On the other hand, Witteveen et al. (2016) demonstrated that SIN3A is expressed throughout the brain in developing mice, with higher levels in the subventricular zone, rostral migratory stream, and olfactory bulb. *In vivo* functional knockdown of *Sin3a* leads to reduced cortical neurogenesis, altered neuronal identity, and aberrant corticocortical projections. In humans, haploinsufficiency of *SIN3A* leads to a broad range of neurodevelopmental disorders, explained by reduced cortical neurogenesis (Witteveen et al., 2016). Altogether, the evidence from patients with WITKOS and animal model studies has allowed to suggest that alterations in cortical expansion would be a direct consequence of *SIN3A* haploinsufficiency, leading to a broad range of neurodevelopmental disorders. An impaired expression in the structures originating the olfactory bulb and GnRH neurons can explain the reproductive and olfactory phenotypes observed in our patient and another case recently reported with WITKOS and Kallmann syndrome (Schnoll et al., 2023).

Pubertal delay, cryptorchidism, and micropenis, which can be due to hypogonadotropic hypogonadism, have been reported in patients with WITKOS due to 15q24 microdeletions (Cushman et al., 2005; Magoulas and El-Hattab, 2012; Schnoll et al., 2023). The shortest region of overlap contains eight disease-related genes: *COX5A*, *CYP11A1*, *EDC3*, *MAN2C1*, *MPI*, *SEMA7A*, *SIN3A*, and *STRA6*. Of them, only *SEMA7A* plays a role in GnRH neuron migration in mice (Messina et al., 2011). However, which of the eight deleted genes present in the shortest region of overlap is responsible for the reproductive phenotype in patients with WITKOS has not been determined. Here, we describe the first female patient with pubertal delay due to Kallmann syndrome with WITKOS and a loss-of-function variant in *SIN3A*. The analysis of the other contiguous seven genes did not reveal any clinically relevant variant. Additionally, no clinically significant variants were found in the 96 candidate genes that we selected, according to the literature evidence linking them with hypogonadotropic hypogonadism. Interestingly, defects in *SIN3A* have recently been associated with syndromic congenital hypogonadotropic hypogonadism in two male patients with WITKOS, one of them with a 550-kb deletion at 15q24.1 and another with a nonsense rare variant in the N-terminal region of SIN3A (Schnoll et al., 2023). Altogether, these data indicate that *SIN3A* haploinsufficiency could underlie the reproductive phenotype in patients with overlapping phenotypes of WITKOS and Kallmann syndrome.

Like in many other complex syndromic disorders with neurodevelopmental and behavior issues, the neurologic symptoms and intellectual disability predominantly capture medical attention and drive patient management, interfering with a wider clinical assessment, which may explain the probable underdiagnosis of pubertal developmental disorders in patients with WITKOS. Indeed, cryptorchidism and micropenis can anticipate pubertal delay in male populations with hypogonadotropic hypogonadism, but may go undiagnosed if not specifically sought. In the female population, the situation is even more difficult given that there are no early clinical signs of hypogonadism before the age of puberty (Palmert and Dunkel, 2012). In the present case, the absence of thelarche and the endocrine compromise of the GnRH axis only at the age of 14 years drove the genetic assessment in search for the etiology of hypogonadotropic hypogonadism. The unexpected result of the WES study and the consequent reverse phenotyping allowed to reach the diagnosis of WITKOS and to explain the short stature, which was the reason for referral to the pediatric endocrinologist, together with the neurodevelopmental phenotype and multiple dysmorphic features. An early etiologic diagnosis is important for the establishment of a multidisciplinary management from an early age, for instance, therapy for neurodevelopmental delay; pharmacological treatment for psychiatric problems and epilepsy; and interventions for cardiac, digestive, and ophthalmologic problems. From the endocrinological standpoint, the surveillance of growth and pubertal onset impact the patient’s quality of life deriving from social adjustment related to the acquisition of secondary sexual characteristics, adult height, and sexual maturity in the less severe cases. Finally, further mechanistic studies will be necessary to elucidate the role of *SIN3A* in the regulation of the hypothalamic–pituitary–gonadal axis.

## Data Availability

The data sets presented in this study can be found in online repositories. The names of the repository/repositories and accession number(s) can be found at: NCBI PRJNA1055058.

## Supplementary material

SUPPLEMENTARY TABLE S1 Analysis and interpretation of results using the TSO sequencing panel (Illumina).

SUPPLEMENTARY TABLE S2 Analysis and interpretation of WES, using the xGen Exome Research Panel v2 capture kit (Integrated DNA Technologies, Coralville, IA, United States).

SUPPLEMENTARY APPENDIX S1 Coverage of target sequences of 69 genes associated with hypogonadotropic hypogonadism in the TSO sequencing panel (Illumina).

SUPPLEMENTARY APPENDIX S2 Coverage of target sequences of the 96 genes associated with hypogonadotropic hypogonadism in the WES analysis.
