# Supplementary material for: Case report: Novel SIN3A loss-of-function variant as causative for hypogonadotropic hypogonadism in Witteveen–Kolk syndrome
Source: Front Genet. 2024 Mar 11;15:1354715. doi: 10.3389/fgene.2024.1354715 (PMC10961356; doi:10.3389/fgene.2024.1354715)
Supplement: Supplementary file 1 [file DataSheet2.pdf]

Appendix 2. Coverage of target sequences

Patient name: WES-001

The following genes are 100% covered (with a depth of at least 20x):
AMH, AMHR2, AXL, BMP4, CCDC141, CHD7, CHL1, CPE, CUL4B, DCAF17, DCC, DUSP6, EBF2, FEZF1, FGF17, FGF8, FGFR1, FLRT3, FSHB, GHSR, GLCE, GLI2, GLI3, GNRH1, GNRHR, HAMP, HDAC8, HESX1, HFE, HS6ST1, IGFALS, IGSF1, IGSF10, IL17RD, JAG1, KAL1, KISS1, KISS1R, KLB, LEP, LEPR, LHX3, LHX4, MC3R, MKRN3, MSX1, NDN, NDNF, NELFCD, NHLH2, NOTCH1, NOTCH2, NR0B1, NR5A1, NSMF, NTN1, OTUD4, OTX2, PAX6, PCSK1, PHIP, PLXNA1, PNPLA6, POLR3A, POLR3B, PROK2, PROKR2, PROP1, PTCH1, RAB18, RBM28, RNF216, SEMA3A, SEMA3C, SEMA3E, SEMA3F, SEMA3G, SEMA7A, SLC29A3, SLC40A1, SLIT2, SMCHD1, SOX10, SOX11, SOX2, SOX3, SPRY4, TAC3, TACR3, TBX3, TCF12, TFR2, TUBB3, WDR11

Certain parts of the following genes are not 100% covered (see details below)

Legend:
- exons covered at 100% (with a depth of at least 20x)
- exons covered at 90-100% (with a depth of at least 20x)
- exons covered at <90% (with a depth of at least 20x)
- exons not covered (with a depth of at least 20x)

LHB 91,3%
NEMF 97,2%

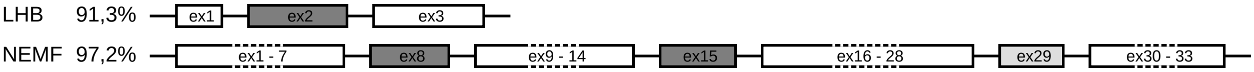

*Using CovReport: Gorokhov M, Cerino M, Mortreux J, Riccardi F, Lévy N, Bartoli M, Krahn M, Gorokhova S. A new tool CovReport generates easy-to-understand sequencing coverage summary for diagnostic reports. Sci Rep. 2020 Apr 10;10(1):6247. doi: 10.1038/s41598-020-63079-4. PMID: 32277129; PMCID: PMC7148332.
