# Supplementary material for: Case report: Novel SIN3A loss-of-function variant as causative for hypogonadotropic hypogonadism in Witteveen–Kolk syndrome
Source: Front Genet. 2024 Mar 11;15:1354715. doi: 10.3389/fgene.2024.1354715 (PMC10961356; doi:10.3389/fgene.2024.1354715)
Supplement: Supplementary file 3 [file DataSheet1.pdf]

Appendix 1. Coverage of target sequences*

Patient name: CH1904224

The following genes are 100% covered (with a depth of at least 20x):
CHL1, CUL4B, FSHB, GNRH1, HAMP, HDAC8, HESX1, KISS1, LHB, MKRN3, OTX2, SEMA3A

Certain parts of the following genes are not 100% covered (see details below)

Legend:
- exons covered at 100% (with a depth of at least 20x)
- exons covered at 90-100% (with a depth of at least 20x)
- exons covered at <90% (with a depth of at least 20x)
- exons not covered (with a depth of at least 20x)

AMH	83,0%	ex1, ex2 - 3, ex4, ex5
AMHR2	97,1%	ex1, ex2, ex3, ex4 - 7, ex8, ex9 - 10, ex11
AXL	94,4%	ex1 - 8, ex9, ex10 - 20
BMP4	81,0%	ex3 - 4
CHD7	95,5%	ex2 - 3, ex4 - 11, ex12, ex13, ex14 - 20, ex21 - 23, ex24 - 30, ex31, ex32, ex33 - 34, ex35 - 37, ex38
CPE	95,2%	ex1, ex2 - 9
DCAF17	99,3%	ex1 - 13, ex14
DCC	98,3%	ex1 - 14, ex15, ex16 - 19, ex20, ex21 - 23, ex24, ex25 - 29
FGF8	89,8%	ex1, ex2 - 3, ex4, ex5, ex6
FGFR1	95,0%	ex2, ex3, ex4, ex5 - 7, ex8, ex9 - 15, ex16, ex17 - 19
GHSR	90,4%	ex1, ex2
GLI2	85,4%	ex2 - 3, ex4, ex5 - 6, ex7, ex8 - 9, ex10, ex11, ex12, ex13 - 14
GLI3	92,4%	ex2, ex3, ex4, ex5, ex6 - 10, ex11, ex12, ex13 - 14, ex15
GNRHR	92,6%	ex1, ex2 - 3
HFE	88,4%	ex1, ex2, ex3 - 4, ex5 - 6
HS6ST1	0,0%	ex2
IGFALS	95,1%	ex1, ex2
JAG1	94,2%	ex1, ex2 - 3, ex4, ex5 - 25, ex26
KAL1	98,6%	ex1, ex2 - 14
KISS1R	92,7%	ex1 - 4, ex5
KLB	92,0%	ex1, ex2 - 3, ex4, ex5
LEP	79,6%	ex2, ex3
LEPR	92,7%	ex3, ex4 - 5, ex6 - 12, ex13, ex14 - 19, ex20
LHX3	88,1%	ex1, ex2, ex3 - 4, ex5, ex6
LHX4	79,1%	ex1, ex2, ex3, ex4, ex5 - 6
MC3R	92,0%	ex1
MSX1	76,4%	ex1, ex2
NDN	88,0%	ex1
NOTCH1	92,7%	ex1, ex2 - 3, ex4, ex5 - 7, ex8 - 9, ex10 - 11, ex12, ex13 - 14, ex15, ex16 - 17, ex18 - 19, ex20 - 21, ex22, ex23 - 24, ex25 - 26, ex27, ex28, ex29 - 33, ex34
NOTCH2	90,7%	ex1 - 3, ex4, ex5 - 6, ex7, ex8 - 10, ex11, ex12, ex13, ex14 - 21, ex22 - 23, ex24, ex25 - 26, ex27 - 28, ex29 - 30, ex31, ex32 - 33, ex34
NR0B1	90,9%	ex1 - 2
NR5A1	87,0%	ex2 - 3, ex4, ex5 - 6, ex7
NSMF	93,8%	ex1, ex2, ex3, ex4 - 16
PAX6	97,6%	ex4 - 12, ex13, ex14

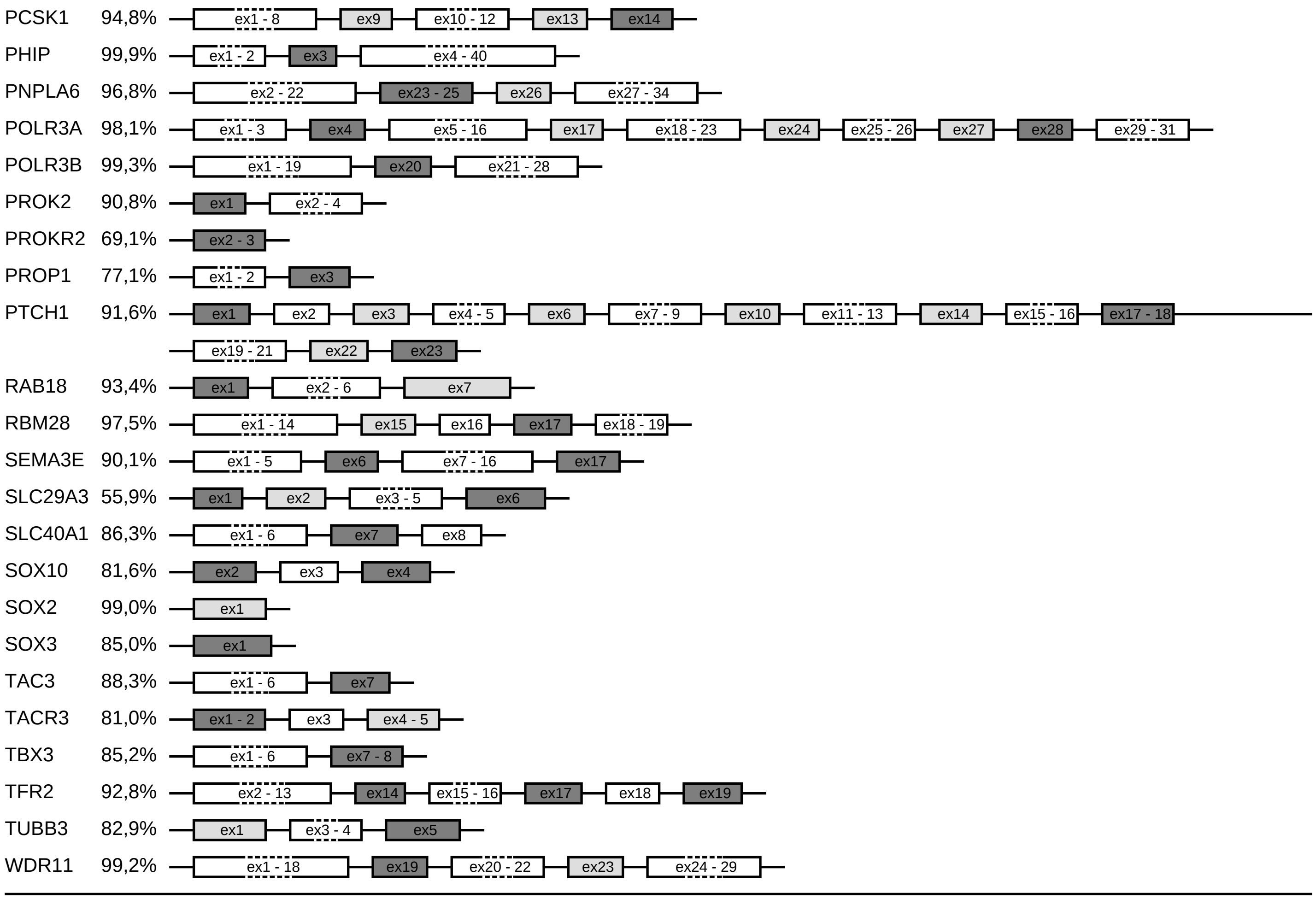

*Using CovReport: Gorokhov M, Cerino M, Mortreux J, Riccardi F, Lévy N, Bartoli M, Krahn M, Gorokhova S. A new tool CovReport generates easy-to-understand sequencing coverage summary for diagnostic reports. Sci Rep. 2020 Apr 10;10(1):6247. doi: 10.1038/s41598-020-63079-4. PMID: 32277129; PMCID: PMC7148332.
